# The Practices of Pancreatic Neck Margin Testing and Their Outcomes after Pancreatoduodenectomy for Pancreatic Ductal Adenocarcinoma

**DOI:** 10.1245/s10434-026-20092-7

**Published:** 2026-07-05

**Authors:** Kimberly P. Woo, William C. Bennett, Jenny H. Chang, Chase J. Wehrle, Noah X. Tocci, Divya Deverakonda, John McMichael, Robert F. Naples, Kathryn A. Stackhouse, Daniel Joyce, Robert Simon, Toms Augustin, Anthony Visioni, R. Matthew Walsh, Nicholas A. Austin, Samer A. Naffouje

**Affiliations:** 1 Department of General Surgery, Cleveland Clinic Foundation, Cleveland, OH USA; 2 Department of General Surgery, Cleveland Clinic Akron General, Akron, OH USA; 3 Department of Radiology, Cleveland Clinic Foundation, Cleveland, OH USA

**Keywords:** Pancreatic ductal adenocarcinoma, Pancreatic neck margin, Pancreatoduodenectomy

## Abstract

**Background:**

The role of checking the pancreatic neck margin with frozen section (PNM-FS) during pancreatoduodenectomy (PD) for pancreatic ductal adenocarcinoma (PDAC) to secure R0 resection and its impact on long-term outcomes remains an area of debate.

**Patients and Methods:**

Patients with PD for PDAC between 2018 and 2023 were included. The primary outcome was pancreatic neck recurrence-free survival (PN-RFS). Secondary outcomes were locoregional recurrence-free survival (LR-RFS), distant recurrence-free survival (D-RFS), and overall survival (OS).

**Results:**

There were 403 patients who met inclusion criteria. PNM-FS was checked in 361 patients (89.6.2%) and returned negative at first attempt in 285 (78.9%). A total of 76 patients had positive PNM-FS; 71 were revised and clearance achieved on repeat sampling in 49 (69.0%). Median PN-RFS in patients with PNM-FS cleared with revision was significantly shorter compared with those with negative PNM-FS at first attempt (16.9 versus 23.1 months; *p* = 0.048) but was not different from patients with PNM-FS not cleared (16.9 versus 16.0 months; *p* = 0.961). LR-RFS and D-RFS were not different between the groups. OS was marginally longer in patients with negative PNM-FS at first attempt (23.9 versus 19.4 versus 16.0 months; *p* = 0.049). Six patients underwent completion total pancreatectomy (TP) for margin clearance, four developed distant recurrence, one locoregional recurrence and two died without disease at 1 and 11 months postoperatively.

**Conclusions:**

PNM-FS is only a prognostic marker. Pursuing PNM-FS clearance does not improve PN-RFS, LR-RFS, D-RFS, or OS. Furthermore, pursuing a completion TP may be associated with shorter OS either from disease progression or surgical morbidity.

**Supplementary Information:**

The online version contains supplementary material available at https://doi.org/10.1245/s10434-026-20092-7.

Management of the pancreatic neck margin (PNM) during pancreatoduodenectomy (PD) for pancreatic ductal adenocarcinoma (PDAC) is crucial for the projected impact of margin status on patients’ recurrence-free survival (RFS) and overall survival (OS). Studies have shown that achieving a negative margin (R0) is associated with improved survival outcome.^1–6^ Patients with an R0 resection of the PNM on permanent section (PS) had a median OS of 21.1 months compared with 13.7 months (*p* < 0.001) for those with a PS positive margin (R1) of the PNM.^7^ Therefore, the prevailing dogma during an oncologic PD is to evaluate the PNM with an intraoperative frozen section (FS). And if the FS reveals a positive margin, follow with resection of additional pancreatic tissue until R0 is achieved.^8^

However, the value of pursuing a negative PNM intraoperatively remains an area of debate. Some studies suggest that clearing the PNM intraoperatively reduces the likelihood of residual tumor and may confer improved survival.^1^ Proponents of this philosophy advocate for proceeding to a total pancreatectomy (TP) if necessary to clear an isolated positive PNM, despite the associated morbidity, justified by the promise of improved OS in return.^2^ On the contrary, other investigations showed no survival benefit with clearing the PNM intraoperatively, suggesting this margin may be indicative of an infiltrative nature of the PDAC, and that furthermore, the biological aggressiveness of the tumor overrides the benefits of achieving a negative margin.^7,9–16^ In addition, resection to achieve R0 status can increase operative time and the risk of complications, which may not be justified if the survival benefit is marginal.^9^

The conception of this current work was sparked by the stark paradox between these two schools of thought regarding PNM management. It is important for pancreas surgeons to ascertain whether an oncologic benefit exists in pursuing a negative PNM in the operating room, as this decision may change the entire operative profile. Furthermore, it can expose patients to increased risk of morbidity and sometimes mortality, which can only be justified by a significantly improved long-term survival outcome. In this study we aim to shed light on the different practices of intraoperative PNM management and their associated outcomes to answer this question.

## Patients and Methods

### Study Design and Inclusion Criteria

This is a retrospective review of adult patients, 18 years and older, who underwent pancreatectomy (PD) for pancreatic ductal adenocarcinoma (PDAC) at the Cleveland Clinic between January 2018 and August 2023. This study was conducted with approval from the Institutional Review Board at the Cleveland Clinic (IRB# 24-907). The Strengthening the Reporting of Observational Studies in Epidemiology (STROBE) reporting guidelines were followed.^17^

### Patient Identification and Data Source

Patients meeting inclusion criteria were identified from the institutional electronic medical record (EMR) using natural language processing. The following search strategy was applied in a stepwise fashion: surgery date between January 2018 and August 2023, diagnosis contains the terms “malignant neoplasm” OR “adenocarcinoma” OR “carcinoma” OR cancer”, procedure contains “pancreatectomy” OR “Whipple”, and pathology report contains “pancreas” AND “adenocarcinoma”. The EMR of patients who were identified from the query were manually reviewed to confirm that all inclusion criteria were met. Surgical pathology reports were reviewed to confirm the tumor histologic type as “pancreatic ductal adenocarcinoma” or a subtype of PDAC. Operative reports were reviewed for the total number of margin revisions performed and whether conversion to completion total pancreatectomy occurred. Patients who underwent upfront planned total pancreatectomy were excluded.

The pathology report was further reviewed to identify the presence or absence of PNM-FS testing and to determine the PNM-FS status and final resection margin status. A margin of less than 1 mm was considered positive and a margin greater than or equal to 1 mm was considered negative. Patients were then divided into six groups on the basis of the intraoperative practices of testing the PNM with FS: (1) PNM-FS negative at first attempt, (2) PNM-FS cleared with revision, (3) PNM-FS cleared with total pancreatectomy (TP), (4) PNM-FS not cleared with FS (remained positive), (5) PNM-FS not checked, final negative, and (6) PNM-FS not checked, final positive.

### Study Outcomes and Statistical Analysis

The primary outcome was pancreatic neck recurrence-free survival (PN-RFS). Secondary outcomes were locoregional recurrence-free survival (LR-RFS), distant recurrence-free survival (D-RFS), and overall survival (OS).

Surveillance imaging demonstrating evidence of recurrence were reviewed by an independent radiologist (N.A.) specializing in abdominal radiology to confirm the findings and determine the category of locoregional recurrence (pancreatic neck versus retroperitoneal versus nodal). The radiographic criteria used were as follows: new or enlarging soft tissue in the surgical bed, persistent soft tissue in the surgical bed with new vascular irregularity, new or enlarging regional lymph nodes without an explainable alternative etiology, and obstruction of the biliopancreatic limb with or without a definable soft tissue mass.

For outcomes analysis, we used the Cox regression analysis to identify independent predictors of survival, Kaplan–Meier method to draw survival plots, and log-rank test to compare outcomes between the groups. Fisher’s exact test was used to compare categorical outcomes between comparison groups. A *p*-value of < 0.0.5 was considered statistically significant throughout the study. All statistical analyses were conducted using SPSS v29 (IBM, Armonk, NY).

## Results

A total of 403 patients were included with a median follow-up and surveillance duration of 19 and 16 months, respectively. Table 1 presents the demographic and clinical characteristics of the selected patient population.

**Table 1.** Summary of the demographic and clinical characteristics of the patient population

N	403
Age	
Mean ± SD	68.0 ± 9.9
Median [IQR]	69 [62–75]
Sex	
Male	215 (53.3%)
Female	188 (46.7%)
Race	
White	327 (81.1%)
Black	61 (15.1%)
Other	15 (3.7%)
Approach	
Open	391 (97.0%)
Robotic	12 (3.0%)
Margin status	
Negative	263 (65.3%)
Pancreatic neck only	16 (4.0%)
Retroperitoneum only	102 (25.3%)
Bile duct only	3 (0.7%)
Multiple	19 (4.7%)
Specific margin status	
Pancreatic neck positive	25 (6.2%)
Retroperitoneum positive	121 (30.0%)
Node status	
Negative	113 (28.0%)
Positive	290 (72.0%)
Examined nodes	
Mean ± SD	22.2 ± 11.0
Median [IQR]	20 [14–28]
Positive nodes	
Mean ± SD	3.0 ± 3.6
Median [IQR]	2 [0–4]
Chemotherapy	
Neoadjuvant	109 (27.0%)
Median neoadjuvant cycles [IQR]	6 [4–6]
Adjuvant	279 (69.2%)
Median adjuvant cycles [IQR]	6 [3–12]
Radiation/chemoradiation	
Neoadjuvant	23 (5.7%)
Median neoadjuvant fractions [IQR]	28 [28–28]
Adjuvant	42 (10.4%)
Median adjuvant fractions [IQR]	28 [28–28]

**Fig. 1:**
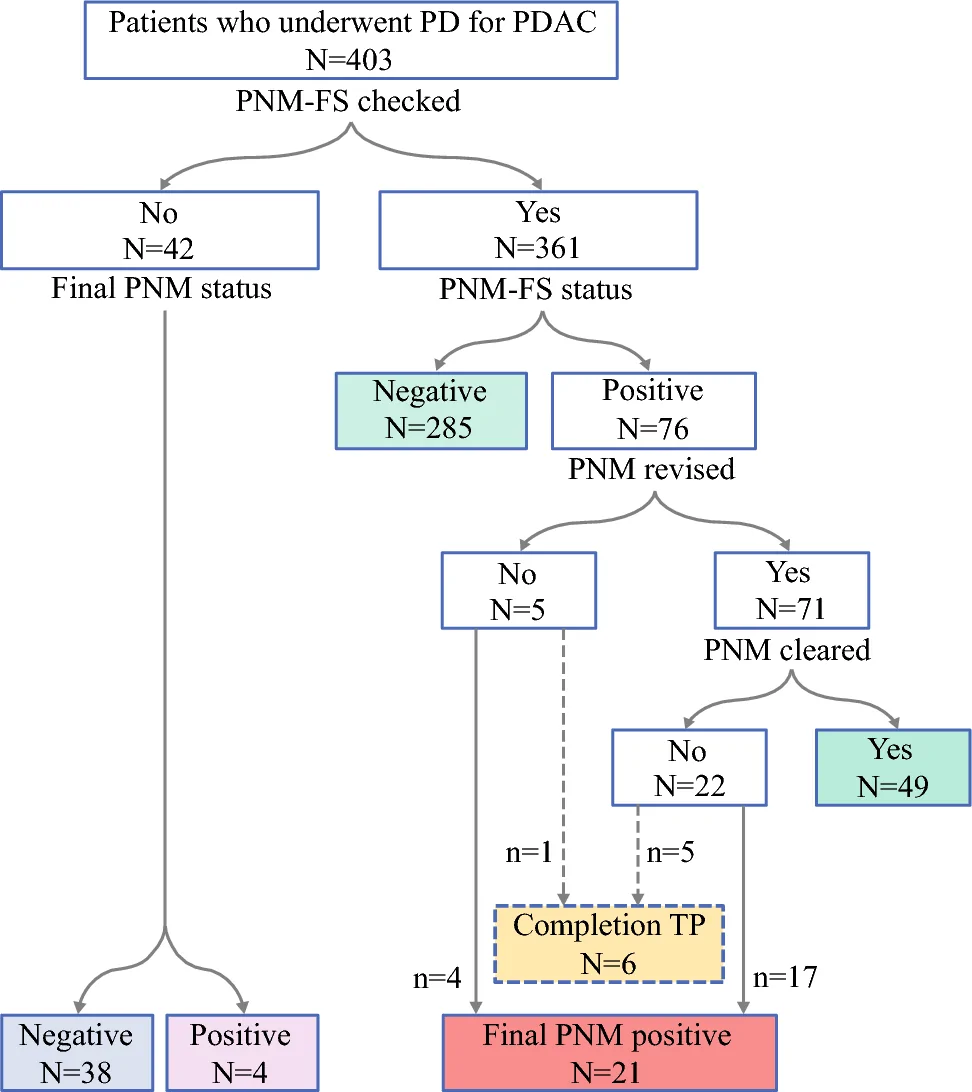
Diagram showing the different PNM-FS practices and management of different scenarios during PD for PDAC at our institution between 2018 and 2023. PD: Pancreaticoduodenectomy; PDAC: Pancreatic Ductal Adenocarcinoma; PNM-FS: Pancreatic Neck Margin Frozen Section, TP: Total Pancreatectomy

PNM-FS was tested in 361 patients (89.6%), whereas 42 (10.4%) completed their PD without testing for PNM-FS intraoperatively. Of those who had PNM-FS checked, the margin returned negative at first attempt in 285 (78.9%). A total of 76 patients had a positive PNM-FS, which was revised in 71, and 5 patients did not undergo PNM-FS revision. In the latter group (i.e., PNM-FS not revised), one patient had a completion TP and 4 completed their PD with a positive PNM-FS. Of the 71 cases with revised PNM-FS, clearance was achieved with repeat sampling in 49 cases (69.0%), whereas 22 patients had persistently positive PNM-FS; 17 patients in the latter group completed their PD with positive PNM, whereas 5 patients received a completion TP. Figure 1 summarizes the scenarios of intraoperative PNM management and outcomes.

The patient population was divided into six groups on the basis of the practices and outcomes previously described in the Methods section. Table 2 summarizes the characteristics of each patient group. The first outcomes analysis included the following three groups: PNM-FS negative (group 1, *n* = 285), PNM-FS cleared with revision (group 2, *n* = 49), and PNM-FS not cleared (group 4, *n* = 21). The second analysis was performed between patients with PNM-FS cleared with TP (group 3, *n* = 6) and PNM-FS not cleared (group 4, *n* = 21).

**Table 2.** Demographic and clinical characteristics of the groups reflecting different practices of intraoperative PNM-FS. IQR: Interquartile Range; PNM-FS: Pancreatic Neck Margin Frozen Section; SD: Standard Deviation

	PNM-FS negative	PNM-FS cleared w revision	PNM-FS cleared w total panc	PNM-FS not cleared	PNM-FS not checked- final negative	PNM-FS not checked- final positive
N	285	49	6	21	38	4
Age (mean ± SD)	67.8 ± 9.9	69.6 ± 8.0	73.0 ± 7.8	67.9 ± 9.3	66.7 ± 12.1	71.3 ± 8.4
Sex						
Male	149 (52.3%)	24 (49.0%)	4 (66.7%)	12 (57.1%)	23 (60.5%)	3 (75.0%)
Female	136 (47.7%)	25 (51.0%)	2 (33.3%)	9 (42.9%)	15 (39.5%)	1 (25.0%)
Race						
White	229 (80.4%)	41 (83.7%)	5 (83.3%)	16 (76.2%)	33 (86.8%)	3 (75.0%)
Black	46 (16.1%)	7 (14.3%)	0 (0.0%)	5 (23.8%)	3 (7.9%)	0 (0.0%)
Other	10 (3.5%)	1 (2.0%)	1 (16.7%)	0 (0.0%)	2 (5.3%)	1 (25.0%)
Approach						
Open	278 (97.5%)	49 (100.0%)	5 (83.3%)	21 (100.0%)	35 (92.5%)	3 (75.0%)
Robotic	7 (2.5%)	0 (0.0%)	1 (16.7%)	0 (0.0%)	3 (7.9%)	1 (25.0%)
Other positive margins	84 (29.5%)	22 (44.9%)	2 (33.3%)	5 (23.8%)	9 (23.7%)	4 (100.0%)
Location of other positive margins						
Retroperitoneum	80 (95.2%)	21 (95.5%)	2 (100.0%)	5 (100.0%)	9 (100.0%)	4 (100.0%)
Bile duct	3 (3.6%)	0 (0.0%)	0 (0.0%)	0 (0.0%)	0 (0.0%)	0 (0.0%)
Gastric	0 (0.0%)	1 (4.5%)	0 (0.0%)	0 (0.0%)	0 (0.0%)	0 (0.0%)
Multiple	1 (1.2%)	0 (0.0%)	0 (0.0%)	0 (0.0%)	0 (0.0%)	0 (0.0%)
Node status						
Negative	86 (30.2%)	10 (20.4%)	1 (16.7%)	6 (28.6%)	9 (23.7%)	1 (25.0%)
Positive	199 (69.8%)	39 (79.6%)	5 (83.3%)	15 (71.4%)	29 (76.3%)	3 (75.0%)
Chemotherapy						
Neoadjuvant	83 (29.1%)	9 (18.4%)	3 (50.0%)	7 (33.3%)	6 (15.8%)	1 (25.0%)
Median neoadjuvant cycles [IQR]	0 [0–4]	0 [0–0]	1.5 [0–4]	0 [0–4.5]	0 [0–0]	0 [0–4.5]
Adjuvant	195 (68.4%)	38 (77.6%)	3 (50.0%)	18 (85.7%)	24 (63.2%)	1 (25.0%)
Median adjuvant cycles [IQR]	4 [0–10]	6 [1–12]	0.5 [0–4.5]	4 [2–6]	2.5 [0–8.5]	0 [0–9]
Radiation/Chemoradiation						
Neoadjuvant	17 (6.0%)	4 (8.2%)	0 (0.0%)	1 (4.8%)	1 (2.6%)	0 (0.0%)
Median neoadjuvant fractions [IQR]	0 [0–0]	0 [0–0]	0 [0–0]	0 [0–0]	0 [0–0]	0 [0–0]
Adjuvant	29 (10.2%)	4 (8.2%)	1 (16.7%)	5 (23.8%)	1 (2.6%)	2 (50.0%)
Median adjuvant fractions [IQR]	0 [0–0]	0 [0–0]	0 [0–0]	0 [0–0]	0 [0–0]	2.5 [0–22.5]

**Fig. 2:**
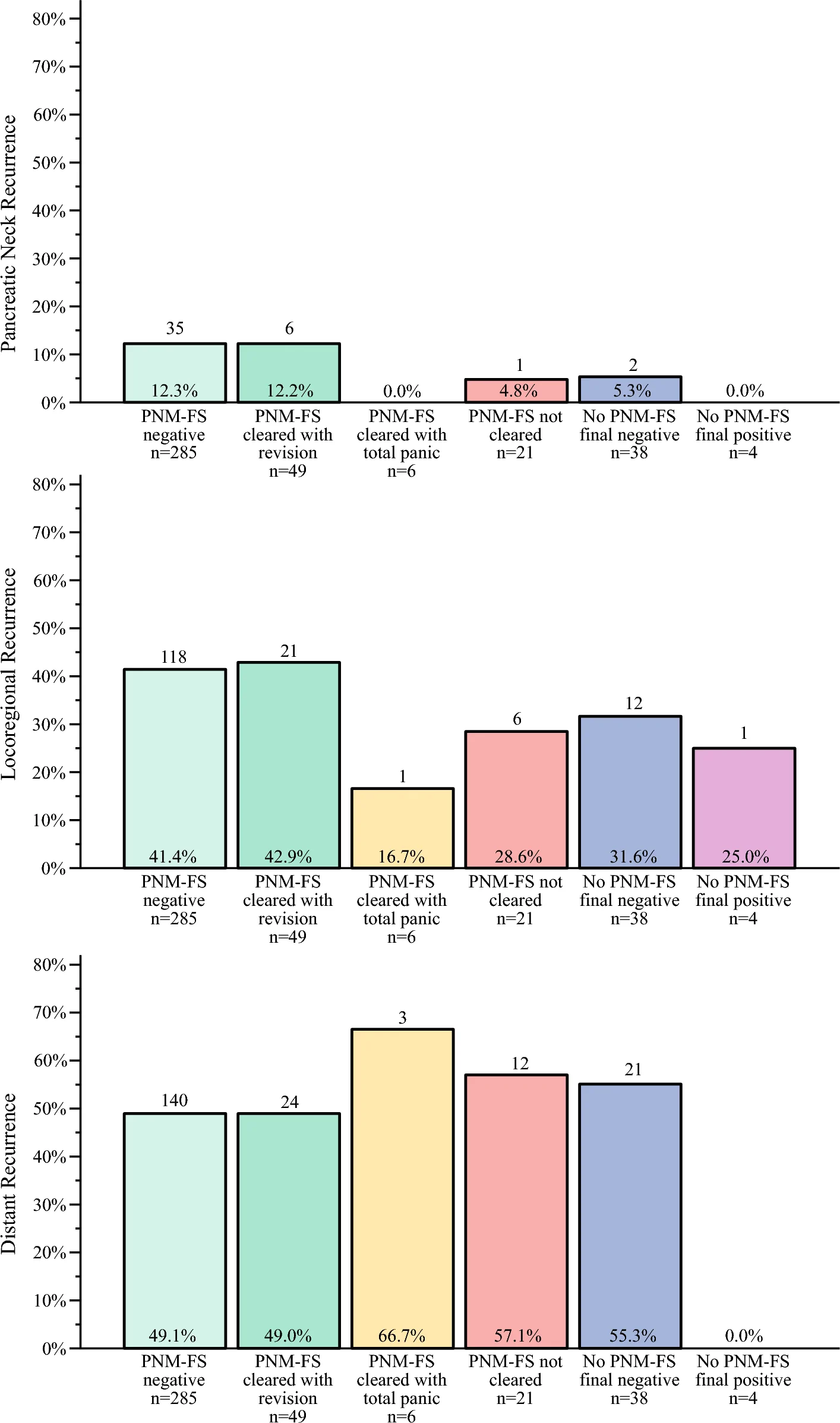
Rates of pancreatic neck, locoregional, and distant recurrence in the groups that reflect different practices of intraoperative PNM management. PNM: Pancreatic Neck Margin

**Fig. 3:**
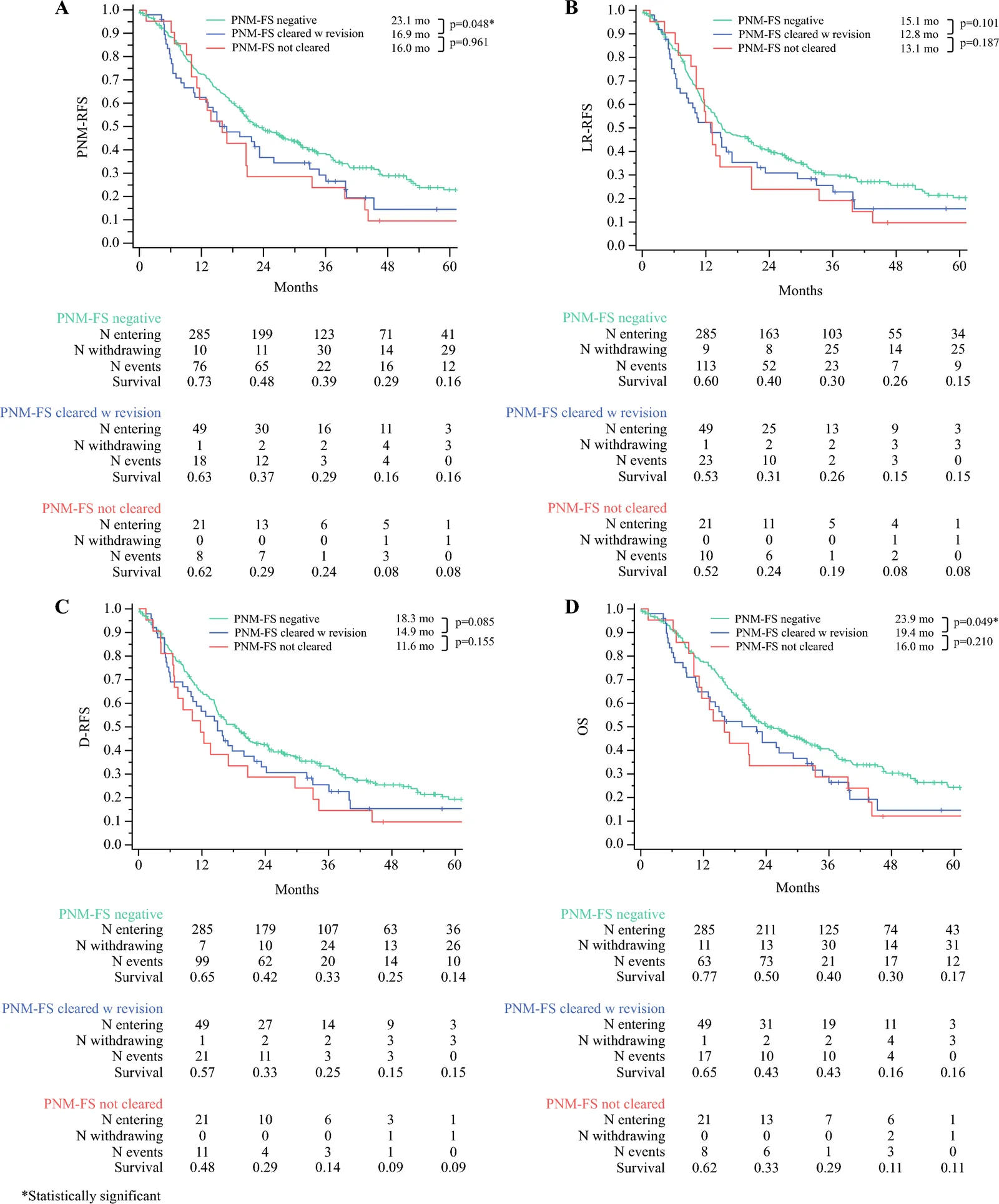
Kaplan Meier analysis for **A** PNM recurrence free survival, **B** locoregional recurrence free survival, **C** distant recurrence free survival, and **D** overall survival in patients who had PNM-FS negative, PNM-FS cleared with revision, and PNM-FS not cleared. FS: Frozen Section; PNM: Pancreatic Neck Margin

Figure 2 demonstrates the rates of pancreatic neck recurrence, locoregional recurrence, and distant recurrence for each of the groups. In group 1, these rates were 12.3%, 41.4%, and 49.1%, respectively. These results were comparable to group 2, where the rates were 12.2%, 42.9%, and 49.0%, respectively (one-way ANOVA, *p* = NS for all), and group 4, with rates of 4.8%, 28.6% and 57.1%, respectively (one-way ANOVA, *p* = NS for all). Moreover, there was no difference between these groups in the rates of neoadjuvant systemic (one-way ANOVA, *p* = 0.658), adjuvant systemic (one-way ANOVA, *p* = 0.657), neoadjuvant radiation (one-way ANOVA, *p* = 0.697), or adjuvant radiation (one-way ANOVA, *p* = 0.621). Kaplan–Meier survival analysis demonstrated a marginally improved PNM-RFS in group 1 compared with groups 2 and 4 (23.1 versus 16.9 versus 16.0 months; *p* = 0.048) with concordant improvement in OS (23.9 versus 19.4 versus 16.0 months; *p* = 0.049) but no difference in LR-RFS (15.1 versus 12.8 versus 13.1 months;* p* = NS) and D-RFS (18.3 versus 14.9 versus 11.6 months; *p* = NS). Figure 3A–D shows the results of Kaplan–Meier survival analyses between groups 1, 2, and 4.

A second survival analysis was performed between groups 3 and 4. The sample size was too small to perform analysis of variance (ANOVA) testing for pre- and postoperative variables. Kaplan–Meier analysis showed no difference in LR-RFS (11.3 versus 13.1 months; *p* = 0.563), D-RFS (10.1 versus 28.5 months; *p* = 0.059), and OS (11.3 versus 16.0 months; *p* = 0.314). Supplementary Fig. 4A–C summarizes these results.

Cox univariable regression analysis was applied to all available variables for prediction of PNM-RFS and concluded age, PNM final status, any other positive margin, positive nodes, and adjuvant systemic therapy as significant predictors. Subsequently, only the latter three factors (other positive margins, positive nodes, and adjuvant systemic therapy) were shown to be significant predictors in the multivariable model. Table 3 presents the results of the univariable-multivariable Cox regression for prediction of PNM-RFS. We then applied a multivariable Cox regression in a backward conditional stepwise approach (inclusion condition *p* < 0.05 and exclusion condition *p* ≥ 0.05) and concluded only four independent predictors of overall survival: age, other positive margins, number of positive nodes, and adjuvant systemic therapy. Final PNM status was excluded in the final block, with *p* = 0.076. Table 4 presents the results of this conditional multivariable Cox regression.

**Table 3.** Cox univariable and multivariable regression analysis for predictors of PNM-RFS. 95% CI: 95% Confidence Interval; PNM: Pancreatic Neck Margin; RFS: Recurrence Free Survival

	Univariable regression		Multivariable regression	
	Hazard Ratio [95% CI]	p	Hazard Ratio [95% CI]	p
Age	1.015 [1.003–1.028]	0.014*	1.011 [0.989–1.025]	0.080
Sex	1.112 [0.881–1.403]	0.373		
Approach	1.001 [0.515–1.946]	0.997		
Final PNM status	1.308 [1.107–2.335]	0.013*	1.452 [0.985–2.141]	0.060
Other positive margins	1.551 [1.213–1.982]	<0.001*	1.557 [1.211–2.002]	<0.001*
Positive nodes	1.732 [1.308–2.294]	<0.001*	1.727 [1.297–2.300]	<0.001*
Neoadjuvant chemotherapy	1.209 [0.939–1.558]	0.141		
Adjuvant chemotherapy	0.518 [0.404–0.663]	<0.001*	0.504 [0.393–0.647]	<0.001*
Neoadjuvant radiation	0.893 [0.530–1.503]	0.670		
Adjuvant radiation	0.917 [0.634–1.235]	0.643		

^*^Statistically significant

**Table 4.** Final block of the backward stepwise conditional Cox regression for predictors of overall survival (inclusion condition p<0.05, exclusion condition p ≥ 0.05). 95% CI: 95% Confidence Interval

	Hazard Ratio [95% CI]	p
Age	1.016 [1.003–1.028]	0.013*
Other positive margins	1.627 [1.263–2.096]	< 0.001*
Number of positive nodes	1.072 [1.044–1.102]	< 0.001*
Adjuvant chemotherapy	0.454 [0.352–0.585]	< 0.001*

^*^Statistically significant

Finally, we selected patients with negative “other margins” (*n* = 279). In this subgroup, 263 patients had negative versus 16 positive final PNM. Pancreatic neck recurrence rates (12.2% versus 12.5%), locoregional recurrence rates (39.2% versus 31.3%), and distant recurrence rates (46.4% versus 56.3%) were not different between the groups (Fisher’s exact test, *p* = NS for all). Supplementary Fig. 5 summarizes the comparison of recurrence rates between negative versus positive final PNM patients with negative “other margins.”

## Discussion

Our analysis suggests that pursuing a positive PNM-FS intraoperatively by performing repeated margin revisions and further pancreatic resections until clearance is achieved does not necessarily convey a meaningful survival benefit. Proceeding to completion TP was particularly associated with worse OS outcomes without a significant difference in oncologic outcomes (LR-RFS, D-RFS, and eventually OS), indicating that the increased morbidity associated with TP (e.g., brittle diabetes, malnutrition, etc.) may not have been justified.

These findings are in line with several other studies that did not support the intraoperative conversion of PNM from FS-R1 into PS-R0 since it did not result in an improvement in OS.^7,12,13,16^ In fact, some of these studies showed worse survival outcomes in patients whose PNM was converted intraoperatively,^7,13^ likely due to the aggressive disease biology and diffuse nature of the tumor rather than a consequence of the intraoperative decision.

However, it is important to examine findings from studies that support this practice with a promise of improved oncologic outcomes. Habib et al. reported that in intraductal papillary mucinous neoplasm (IPMN)-derived PDAC, high-grade dysplasia or invasive cancer at the PNM is associated with poorer outcomes, and converting such high-risk margins to low-risk margins (low-grade or no dysplasia) with a significant improvement in OS.^4^ The discrepancy between their findings and ours likely stems from fundamental differences in patient populations and disease biology. IPMN-associated cancers follow a stepwise dysplastic progression and generally exhibit less aggressive behavior than de novo PDAC, making them more responsive to local clearance. In contrast, conventional PDAC, as studied in our cohort, is typically characterized by diffuse microscopic spread and early systemic dissemination, such that margin revision may not meaningfully alter their oncologic trajectory. Moreover, Habib et al. used a granular margin classification on the basis of dysplasia grade rather than a binary R0/R1 system, and their study population may have included patients with more indolent tumors and limited disease burden. Together, these biological and methodological distinctions likely explain why intraoperative neck margin revision conferred survival benefit in their IPMN-derived cohort but not in ours. Additionally, median OS in their patients with negative margins was reported as 65 months, which is notably longer than the typical survival expectations for pancreatic cancer. This extended survival suggests that achieving negative margins may be associated with more favorable tumor biology.^11^

A meta-analysis by Birgin et al. also reported that achieving FS-R0 during Whipple surgery is associated with improved OS.^3^ This analysis carried a significant heterogeneity in patient selection, tumor biology, and adjuvant treatment protocols between the included studies which could have contributed to the differing outcomes. Finally, a study by Zhang et al. reported that complete tumor extirpation via either en-bloc or non-en-bloc resection based on FS analysis was associated with improved OS compared with incomplete resection, but concerns were raised about the lack of a consensus R0 definition since they used “*no tumor on ink*” as their negative margin, which complicates comparisons across studies.^5^

Regarding the question of completion TP solely to clear a positive PNM, our analysis does not support that practice on the basis of comparable D-RFS and reduced OS outcomes in patients who followed that path, albeit the number in that group was small (group 3, *n* = 6). Schmidt et al. conducted a study analyzing the impact of total pancreatectomy (TP) versus subtotal pancreatectomy in patients with isolated positive PNM and found that patients with R0 after TP had improved OS compared with those with subtotal pancreatectomy and R1.^2^ Their study may be influenced by selection bias and a lack of consideration for modern systemic therapies since it was conducted in an era when chemotherapy regimens were less effective, making surgical margin clearance a more critical factor in survival. However, the study does not account for the significant morbidity of TP, which can delay or limit chemotherapy administration, ultimately negating any potential benefit from achieving R0 resection.^11^ Additionally, with advancements in systemic therapy, particularly FOLFIRINOX and gemcitabine-based regimens, survival is now more dependent on systemic disease control rather than margin status alone.^18,19^ While the analysis by Zhang et al. first reported improved survival benefit associated with margin clearance,^5^ their recently published reappraisal using a contemporary cohort of patients found that additional resection in post-neoadjuvant PD did not demonstrate survival or recurrence benefit.^20^

The advantage of our study in addressing the question of intraoperative PNM status is the granularity in assessing the specific outcome of PNM recurrence (i.e., recurrence at the transected margin or at the pancreaticojejunostomy), a critical yet previously unexamined outcome. Unlike prior studies that broadly assess OS or local recurrence without specifying the exact location, our work precisely differentiates true pancreatic neck recurrence from regional and retroperitoneal recurrences, which may stem from distinct biological mechanisms. This distinction is crucial because prior studies reporting on local recurrence fail to specify whether recurrence occurred at the pancreatic neck or other anatomical sites, limiting their relevance to surgical decision-making regarding the PNM. Furthermore, we employed a dedicated radiologic confirmation to validate recurrence patterns, ensuring accuracy and eliminating potential misclassification. By focusing on margin-specific recurrence, our study challenges conventional assumptions about the oncologic impact of resecting a positive pancreatic neck margin. This level of detail surpasses previous research, offering a more precise and clinically relevant evaluation of margin clearance and its true impact on disease progression and patient outcomes.

## Conclusions

Our analysis suggests that PNM-FS is only a prognostic marker. Pursuing pancreatic neck clearance with margin revision does not imply a difference in PN-RFS, D-RFS, or OS. Furthermore, pursuing a completion TP to clear the margin may be associated with shorter OS either due to the infiltrative and aggressive nature of the tumor or the surgical morbidity associated with TP.

## Supplementary Information

Below is the link to the electronic supplementary material.

Supplementary file1 (DOCX 4150 KB)
