# Supplementary material for: The Practices of Pancreatic Neck Margin Testing and Their Outcomes after Pancreatoduodenectomy for Pancreatic Ductal Adenocarcinoma
Source: Ann Surg Oncol. 2026 Jul 5;33(10):9344–52. doi: 10.1245/s10434-026-20092-7 (PMC13585785; doi:10.1245/s10434-026-20092-7)
Supplement: Supplementary file 1 [file 10434_2026_20092_MOESM1_ESM.docx]

**Supplemental Figure 4**. Kaplan Meier analysis for (**A**) locoregional recurrence free survival, (**B**) distant recurrence free survival, and (**C**) overall survival in patients who had PNM-FS cleared with TP and PNM-FS not cleared. **FS**: Frozen Section; **PNM**: Pancreatic Neck Margin; **TP**: Total Pancreatectomy.

*Statistically significant

**Supplemental Figure 5**. Rates of pancreatic neck, locoregional, and distant recurrence in patients with a final PNM-negative vs. PNM-positive and no other positive margins in the Whipple specimen. **PNM**: Pancreatic Neck Margin.
